# Loss of Kindlin-1 Causes Skin Atrophy and Lethal Neonatal Intestinal Epithelial Dysfunction

**DOI:** 10.1371/journal.pgen.1000289

**Published:** 2008-12-05

**Authors:** Siegfried Ussar, Markus Moser, Moritz Widmaier, Emanuel Rognoni, Christian Harrer, Orsolya Genzel-Boroviczeny, Reinhard Fässler

**Affiliations:** 1 Department of Molecular Medicine, Max-Planck Institute of Biochemistry, Martinsried, Germany; 2 Division of Neonatology, Perinatal Center, Children's Hospital, Ludwig-Maximilians University, Munich, Germany; Medical Research Council Human Genetics Unit, United Kingdom

## Abstract

Kindler Syndrome (KS), characterized by transient skin blistering followed by abnormal pigmentation, skin atrophy, and skin cancer, is caused by mutations in the FERMT1 gene. Although a few KS patients have been reported to also develop ulcerative colitis (UC), a causal link to the FERMT1 gene mutation is unknown. The FERMT1 gene product belongs to a family of focal adhesion proteins (Kindlin-1, -2, -3) that bind several β integrin cytoplasmic domains. Here, we show that deleting Kindlin-1 in mice gives rise to skin atrophy and an intestinal epithelial dysfunction with similarities to human UC. This intestinal dysfunction results in perinatal lethality and is triggered by defective intestinal epithelial cell integrin activation, leading to detachment of this barrier followed by a destructive inflammatory response.

## Author Summary

Mutations in FERMT1, coding for the Kindlin-1 protein, cause Kindler Syndrome in humans, characterized by skin blistering, atrophy, and cancer. Recent reports showed that some Kindler Syndrome patients additionally suffer from ulcerative colitis. However, it is unknown whether this is caused by loss of Kindlin-1 or by unrelated abnormalities such as infections or additional mutations. We ablated the Fermt1 gene in mice to directly analyze the pathological consequences and the molecular mode of action of Kindlin-1. Kindlin-1–deficient mice develop a severe epidermal atrophy, but lack blisters. All mutant mice die shortly after birth from a dramatic, shear force-induced detachment of intestinal epithelial cells followed by a profound inflammation and organ destruction. The intestinal phenotype is very similar to, although more severe than, the one observed in Kindler Syndrome patients. In vitro studies revealed that impaired integrin activation, and thus impaired adhesion, to the extracellular matrix of the intestinal wall causes intestinal epithelial cell detachment. Therefore, we demonstrate that intestinal epithelial cells require adhesive function of integrins to resist the shear force applied by the stool. Furthermore, we provide evidence that the colitis associated with Kindler Syndrome is caused by a dysfunction of Kindlin-1 rather than by a Kindlin-1–independent event.

## Introduction

Kindler Syndrome (KS; OMIM:173650) is a rare, recessive genodermatosis caused by mutations in the FERMT1 gene (C20ORF42/KIND1) [1],[2]. KS patients suffer from varying skin abnormalities that occur at distinct phases of their life [3]. Skin blisters develop and disappear after birth, followed by skin atrophy, pigmentation defects and finally skin cancer. The severity of the individual symptoms varies extensively among individual patients. FERMT1 mutations are distributed along the entire gene and can give rise to different truncated Kindlin-1 proteins [4]. Interestingly, the different courses of the KS cannot be linked to mutations within specific regions of the FERMT1 gene [3] suggesting that additional environmental and/or genetic factors contribute to the disease course.

Kindlin-1 belongs to a novel family of cytoplasmic adaptor proteins consisting of three members (Kindlin-1-3) [5]. Kindlins are composed of a central FERM (band 4.1, ezrin, radixin, moesin) domain, which is disrupted by a pleckstrin homology (PH) domain. They localize to cell-matrix adhesion sites (also called focal adhesions, FAs) where they regulate integrin function. In line with the role of Kindlins in integrin function, keratinocytes from KS patients and Kindlin-1-depleted keratinocytes display impaired cell adhesion and delayed cell spreading [6],[7]. The mechanism how Kindlin-1 regulates integrin function is not understood and controversial. Kindlin-2 (Fermt2) and Kindlin-3 (Fermt3) were shown to bind to the membrane distal NxxY motif of β1 (Itgb1) and β3 (Itgb3) integrin cytoplasmic domains. This binding, in concert with Talin (Tln1) recruitment to the membrane proximal NPxY motif, leads to the activation (inside-out signaling) of β1 and β3 class integrins enabling them to bind to their ligands. Following ligand binding, Kindlin-2 and Kindlin-3 stay in matrix adhesion sites where they link the ECM to the actin cytoskeleton by recruiting ILK and Migfilin (Fblim1) to FAs (outside-in signaling). Consistent with this adaptor function of Kindlins, keratinocytes from KS patients and keratinocytes depleted of Kindlin-1 display impaired cell adhesion and delayed cell spreading [6],[7]. Importantly, however, Kindlin-1 was reported to have different properties than Kindlin-2 and -3, since it was shown to bind like Talin to the proximal NPxY motif of β1 integrin tails [6].

The Kindlins have a specific expression pattern. Kindlin-1 is expressed in epithelial cells, while Kindlin-2 is expressed almost ubiquitously. They are both found at integrin adhesion sites and/or cadherin-based cell-cell junctions. Kindlin-3 is exclusively expressed in hematopoietic cells, where it controls a variety of functions ranging from integrin signaling in platelets [8] to stabilizing the membrane cytoskeleton in mature erythrocytes [9].

Although the FERMT1 gene is expressed in epithelial cells of almost all tissues and organs [5], only abnormalities of the skin and the oral mucosa are associated with KS. Recently it has been reported, however, that some KS patients also develop ulcerative colitis (UC) [4],[10],[11], which together with Crohn's disease belongs to idiopathic inflammatory bowel disease.

UC usually occurs in the second or third decade of life, although the incidence in pediatric patients is steadily rising [12],[13]. UC is restricted to the colon and is characterized by superficial ulcerations of the mucosa. It is currently believed that the mucosal ulcerations are triggered by the release of a complex mixture of inflammatory mediators leading to severe inflammation and subsequent epithelial cell destruction [12]. In line with this paradigm a large number of murine colitis models occur when the innate or adaptive immune response is altered [12]. Genetic linkage analysis in man led to the identification of several susceptibility loci [14],[15].

In line with the UC disease course most of the KS patients develop their first UC symptoms in adulthood. Interestingly, however, one of them suffered from a severe neonatal form of UC and was diagnosed with a null mutation in the FERMT1 gene after developing trauma-induced skin blistering [10]. Since only a few KS patients were reported to develop intestinal symptoms, it is currently debated whether UC development in these patients is directly linked to FERMT1 gene mutations or secondarily caused by a microbial infection or an abnormal inflammatory response.

In this study we directly investigated the role of Kindlin-1 in vivo by generating mice carrying a constitutive null mutation in the Kindlin-1 gene. We demonstrate that Kindlin-1 deficient mice develop skin atrophy and a lethal intestinal epithelial dysfunction, resembling the reported UC in KS patients. The intestinal epithelial dysfunction is caused by defective intestinal epithelial integrin activation leading to extensive epithelial detachment followed by a severe inflammatory reaction.

## Results

### Loss of Kindlin-1 Leads to a Lethal Intestinal Epithelial Dysfunction

**Figure 1:**
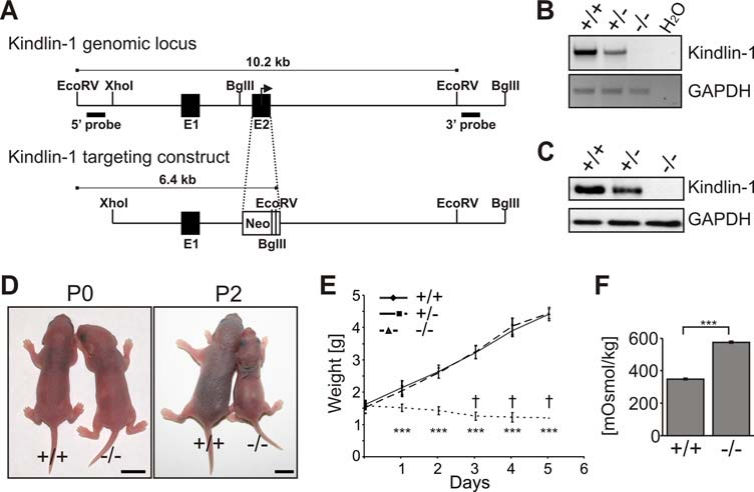
Loss of Kindlin-1 results in early postnatal lethality. (A) The Fermt1 gene was disrupted by replacing the ATG start codon containing exon 2 with a neomycin resistance cassette. (B) Kindlin-1 mRNA levels were determined by PCR from cDNAs derived from P3 kidneys. (C) Loss of Kindlin-1 protein was confirmed by western blotting in colonic IEC lysates. (D) Pictures from newborn (P0) and two day old mice (P2). Scale bars represent 5mm. (E) Weight curve of Kindlin-1^−/−^ (n = 8) and control littermates (+/+; n = 8; +/−; n = 9) where a † indicates when mice died. *** indicates a P-value <0.0001. Error bars show standard deviations. (F) Osmolarity of P2 Kindlin-1^−/−^ and control (+/+) urine (n = 3 per genotype). Error bars show standard deviations. *** indicates a P-value <0.0001.

To unravel the consequences of loss of Kindlin-1 in vivo, we established a mouse strain with a disrupted Fermt1 gene, leading to a complete loss of Kindlin-1 mRNA and protein (Figure 1A–C). Heterozygous Kindlin-1 mice (Kindlin-1^+/−^) had no phenotype and were fertile. Kindlin-1-deficient mice (Kindlin-1^−/−^) were born with a normal Mendelian ratio (29.6% +/+; 44.1% +/−; 26.3% −/−; n = 203 at P0) and appeared normal at birth. Two days postnatal (P2), all Kindlin-1^−/−^ mice analyzed so far were dehydrated (Figure 1D), failed to thrive (Figure 1E) and died between P3-P5 (Figure 1E). Blood glucose and triglyceride levels of Kindlin-1^−/−^ mice were normal suggesting a normal absorption of nutrients in the small intestine (Figure S1). Their urine showed an increased osmolarity and protein content further pointing to severe dehydration (Figure 1F, Figure S2A and B). Histology of Kindlin-1^−/−^ kidneys at P3 revealed normal morphology of glomeruli and tubular systems (Figure S2C). Thus, these findings suggest that the peri-natal lethality is not caused by a renal dysfunction.

**Figure 2:**
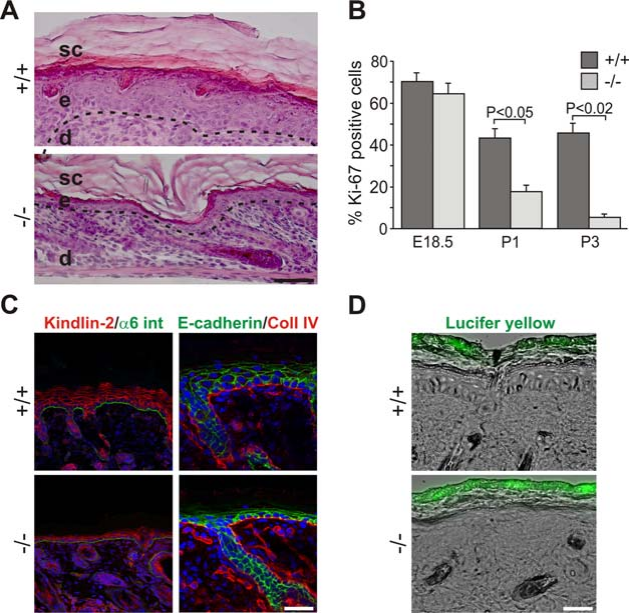
Atrophy and reduced proliferation in Kindlin-1^−/−^ skin. (A) H&E stainings from P3 backskin show severe epidermal atrophy in Kindlin-1^−/−^ mice. The BM is indicated by a dashed line and separates the epidermis (e) from the dermis (d). sc: stratum corneum. Scale bar indicates 50 µm. (B) Percentage of Ki67-positive interfollicular keratinocytes, indicating proliferating cells, at different ages (n = 7 per genotype). Error bars show standard errors of the mean. (C) Kindlin-2 (red; co-stained with α6 integrin (α6 int; in green) and E-cadherin (green, co-stained with Collagen IV (Coll IV) in red) staining of P3 backskin sections. Nuclei are shown in blue. Scale bar indicates 10 µm. (D) FITC-Lucifer yellow stain of P3 backskin overlaid with DIC. Lack of lucifer yellow dye penetration shows normal skin barrier in Kindlin-1^−/−^ mice. Scale bar indicates 50 µm.

Next we analyzed whether skin abnormalities caused the perinatal lethality. Although Kindlin-1^−/−^ mice showed features of KS like skin atrophy and reduced keratinocyte proliferation (Figure 2A and B), adhesion of basal keratinocytes to the basement membrane (BM) was unaltered (Figure 2A and Figure S3). Histology of backskin sections from different developmental stages revealed normal keratinocyte differentiation (Figure S4), normal development of the epidermal barrier (Figure S5A and Figure 2C and D), and comparable epidermal thickness at E18.5 and P0 (Figure S5B). In line with the progressing proliferation defect quantified by the number of keratinocytes positive for the cell cycle marker Ki67 (Mki67), a reduction of the epidermal thickness was first observed at P1 (Figure S5B). Interestingly despite the mild in vivo phenotype, Kindlin-1^−/−^ keratinocytes displayed severe adhesion and spreading defects in culture (Figure S6A and B) further indicating that Kindlin-1^−/−^ keratinocytes from mouse and man display similar defects [7].

**Figure 3:**
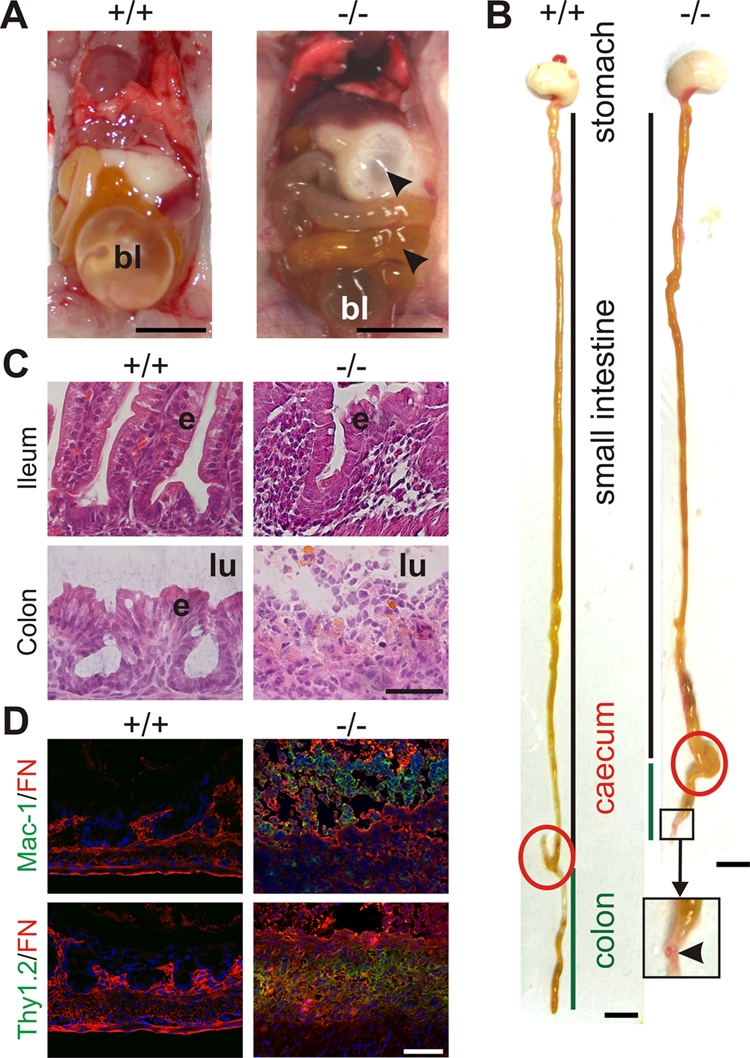
Severe inflammation and epithelial detachment in Kindlin-1^−/−^ colon. (A) Opened abdomen with intestine from P2 mice. Arrowheads indicate air in the stomach and small intestine of Kindlin-1^−/−^ mice. bl; bladder. Scale bar represents 5 mm. (B) Whole gut preparations from P2 mice. Scale bar represents 5 mm. Arrowhead indicates a stricture in the distal colon. The caecum is highlighted with a red circle and the colon is marked with a green line. (C) H&E staining of P3 colon and ileum. Kindlin-1^−/−^ mice show complete absence of colonic epithelium (e), exposure of the submucosa to the intestinal lumen (lu) and severe inflammation in colon and ileum. Scale bar represents 50 µm. (D) Macrophage and granulocyte infiltrations in P3 colon shown with Mac-1 staining in green. T-cell infiltrates in P3 colon shown with Thy1.2 staining in green. Fibronectin (FN) is stained in red. Scale bar represents 50 µm.

These results indicated that another defect is responsible for their perinatal lethality. When the stomach and the intestine of Kindlin-1^−/−^ mice were examined, they were swollen and filled with milk and gas (Figure 3A) suggesting severe intestinal dysfunction might be the cause of death. The presence of milk in the stomach together with the normal histology of the oral and esophageal mucosa suggested that the lethality is not caused by impaired feeding (Figure S7). At P2, the terminal ileum and colon were shortened and swollen and strictures were evident in the distal colon, which are signs of acute inflammation (Figure 3B). By P3, when the majority of mutant mice were dying, more than 80% of the colonic epithelium was detached (Figure 3C and Figure S8A–C), became apoptotic (Figure S9) and infiltrated by macrophages, granulocytes (Mac-1; Itgam staining) and T cells (Thy staining) (Figure 3D). The shortened colon was neither a consequence of increased apoptosis, which was only seen in detached epithelium, nor a result of reduced intestinal epithelial cell (IEC) proliferation (Figure S9).

**Figure 4:**
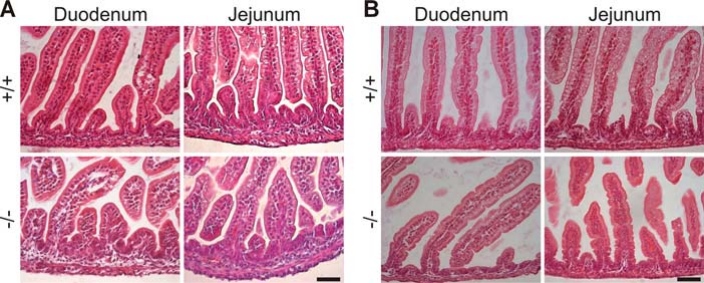
Normal duodenum and jejunum in Kindlin-1^−/−^ mice. H&E stainings of (A) P1 and (B) P3 duodenal and jejunal sections reveal a normal histology of the Kindlin-1^−/−^ small intestine. Scale bar represents 50 µm.

The epithelial detachment and severe inflammation extended into the ileum (Figure 3C). In contrast, the proximal small intestine (duodenum and jejunum) had no evidence of IEC detachment or inflammation (Figure 4). The phenotype of Kindlin-1^−/−^ mice for the most part phenocopied the intestinal disease observed in the patient with a complete loss of Kindlin-1[10].

### Kindlin-1 Is Required for Intestinal Epithelial Cell Adhesion

**Figure 5:**
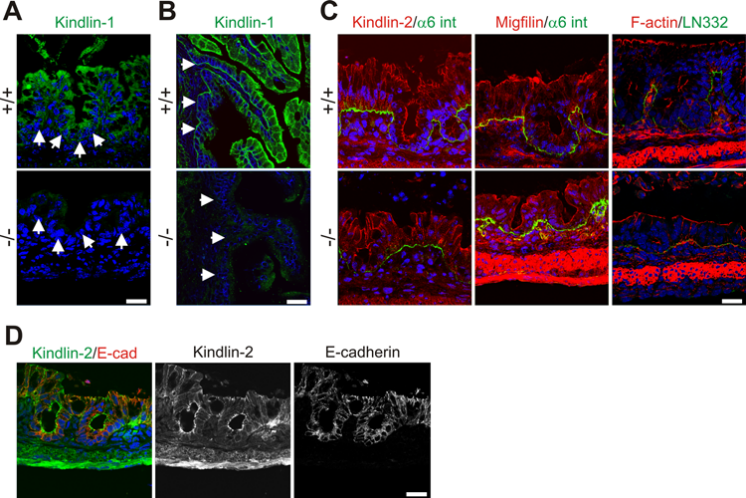
Kindlin-1 localization in mouse intestine. (A) Immunofluorescence staining of Kindlin-1 in neonatal colon. Arrows indicate BM. (B) Immunofluorescence staining of P1 ileum for Kindlin-1. Arrows indicate the BM. (C) Immunofluorescence staining of P1 colon for Kindlin-2, Migfilin, F-actin (red), α6 integrin (α6 int; green) and Laminin-332 (LN332; green). (D) Immunofluorescence staining of colon for Kindlin-2 (green) and E-cadherin (E-cad; red). All scale bars represent 10 µm.

To define the cell type affected by loss of Kindlin-1 we localized Kindlin-1 in the normal intestine by immunostaining. Similar to the situation in man [4], Kindlin-1 is present throughout the cytoplasm of IECs of the colon and at the basolateral sites of both IECs of the colon (Figure 5A) and the small intestine (Figure 5B). The anti-Kindlin-1 polyclonal antibody produced some weak unspecific background signals in the intestinal mesenchyme of wild type and Kindlin-1^−/−^ mice (Figure 5B). Kindlin-2 was exclusively found in cell-cell contacts and did not change its distribution in the absence of Kindlin-1 (Figure 5C and D). Focal adhesion (FA) components such as Talin and Migfilin as well as filamentous actin (F-actin) were expressed normally in Kindlin-1^−/−^ colonic epithelium that was still adhering to the BM (Figure 5C and data not shown).

**Figure 6:**
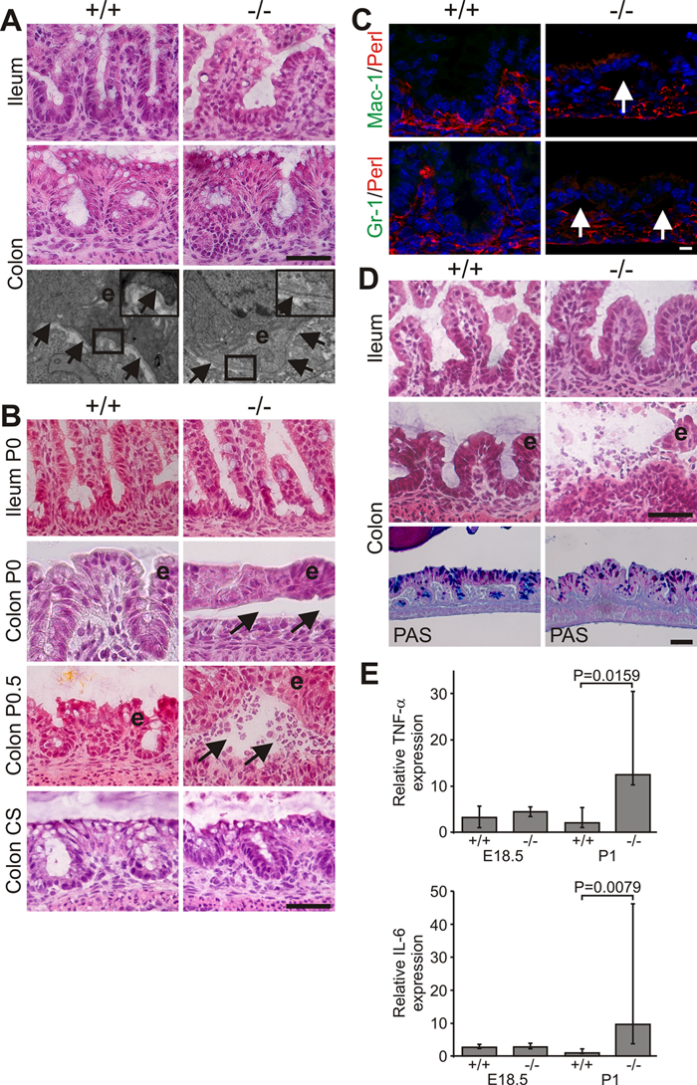
Progressive epithelial dysfunction in Kindlin-1^−/−^ mice. (A) Normal morphology of IECs and BM at E18.5. Shown are H&E stainings of the ileum and colon and electron microscopy pictures at 12000× magnification from the colon. The boxed enlargement shows the BM, e: epithelium. Arrows point to the BM. (B) Colonic IEC (e) detachment at P0 (Colon P0, around 5–6 hours after birth) that becomes infiltrated by immune cells around 12 hours after birth (Colon P0.5). In mice delivered by Caesarean section (CS) and kept unfed for 7 hours no epithelial cell detachment can be observed. Arrows indicate blister. (C) IEC detachment but no macrophage (Mac-1) and granulocyte (GR-1) infiltrations at P0 (Mac-1 and GR-1 in green; Perlecan (Perl) indicating BM in red). Arrows indicate IEC detachment. (D) Immune cell infiltrations in the lumen of the colon and floating epithelial sheets (e) in the colonic lumen at P1. PAS staining shows reduced goblet cell mucins in Kindlin-1^−/−^ colonic epithelium. Scale bars in A, B and D represent 50 µm and in C 10 µm. (E) Median of Real Time PCR results from whole colon mRNA at E18.5 (n = 2 per genotype) and P1 (n = 5 per genotype) for TNF-α and IL-6. Error bars show range. The P value was determined using a Mann-Whitney test.

Next we determined the time point when mutant mice began developing intestinal abnormalities. At E18.5 the ileum and colon of Kindlin-1^−/−^ mice were histologically normal and electron microscopy revealed an intact epithelium and basement membrane (BM) (Figure 6A). Shortly after birth (P0), wild-type and Kindlin-1^−/−^ mice began to suckle and accumulated milk in their stomachs. Within the first hours after birth nursed Kindlin-1^−/−^ mice contained colostrum in the intestinal lumen and displayed extensive epithelial cell detachment (Figure 6B; see Colon P0) without infiltrating immune cells (Figure 6B and C) in the distal colon. No epithelial detachment occurred when Kindlin-1^−/−^ mice were delivered by Caesarean section and incubated in a heated and humidified chamber for up to 7 hours (Figure 6B Colon CS) indicating that mechanical stress applied by stool caused IEC detachment. However, inflammatory infiltrates were clearly visible between the detached epithelial cells and the underlying mesenchyme at around 12 hours after birth in fed mice (Figure 6B; see Colon P0.5) and further increased during the following day (Figure 6D). The steady immune cell infiltrate was accompanied with increased expression of the proinflammatory cytokines TNF-α (Tnf) and IL-6 (Il6) and a reduction in goblet cell mucins (Figure 6D and E). The wide range of TNF-α and IL-6 expression levels in Kindlin-1^−/−^ mice likely reflects the different severities of inflammation at the time tissues were prepared.

Although inflammation extended into the ileum at P2 and P3 (Figure 3B and C), the epithelial cells of the ileum were never detached suggesting that Kindlin-1^−/−^ mice develop a so-called backwash ileitis caused by stool “washed back” from the colon into the ileum [13]. These analyses revealed that the epithelial detachment begins at P0 in the distal parts of the colon and subsequently expands proximally.

### Kindlin-1 Controls Activation of Integrins

**Figure 7:**
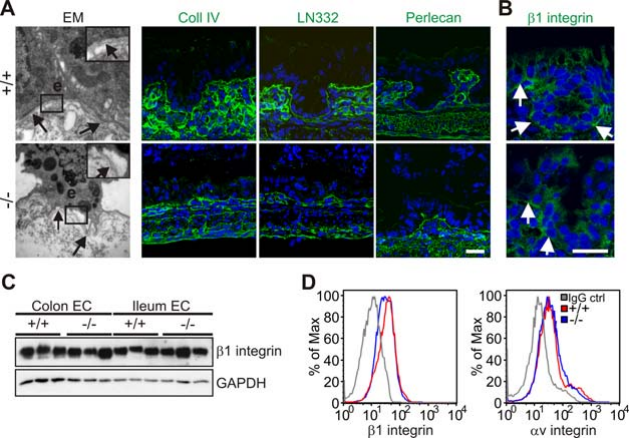
Normal BM composition and integrin localization in P1 colon. (A) Electron micrograph at 12000× magnification shows detachment of colonic IECs from the BM at P1. Arrows point to the BM. The boxed enlargement shows the BM, e: epithelium. Cryo-sections from the colon of P1 Kindlin-1^+/+^ and Kindlin-1^−/−^ mice stained for Collagen IV (Coll IV), Laminin-332 (LN332) and Perlecan. The staining of them shows a normal distribution and localization in Kindlin-1^−/−^ colon. Scale bar represents 10 µm. (B) β1 integrin staining of P1 colon. Arrows indicate the BM. Scale bar represents 10 µm. (C) Western blot from IECs for β1 integrin. (D) β1 and αv integrin FACS profile on primary IECs.

An important question is how Kindlin-1 deficiency leads to detachment of intestinal epithelial cells. One potential explanation could be loss of support by a disrupted BM as reported for skin of KS patients [1],[2],[3]. Moreover, it is known that BM digestion and epithelial detachment in inflammatory bowel disease (IBD) can be triggered via the secretion of MMPs by epithelial and/or infiltrating immune cells [17]. This possibility could be excluded, since Kindlin-1^−/−^ mice at P1 showed a continuous BM with all major components present, both in areas of the colon where IECs were still adherent as well as in areas where IECs were detached (Figure 7A). Interestingly, also the skin of Kindlin-1^−/−^ mice showed a normal BM distribution by immunostaining (Figure S3).

**Figure 8:**
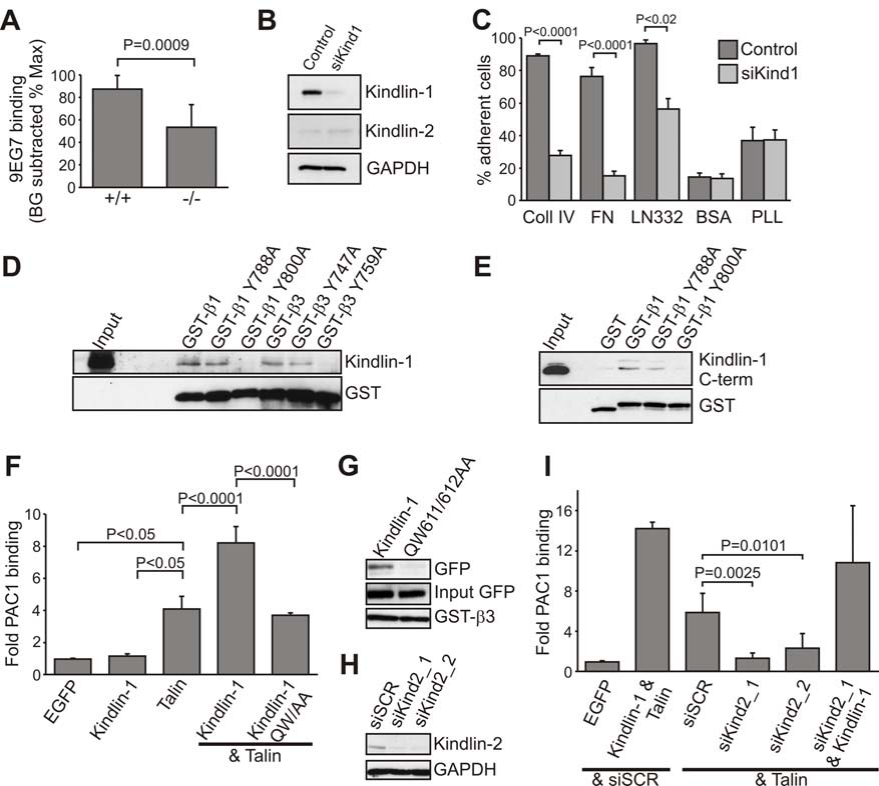
Kindlin-1 association with β integrins is required for Talin-mediated integrin activation. (A) Kindlin-1 IECs display significantly reduced 9EG7 binding (active β1 integrin). The 9EG7 binding was quantified by subtracting background (BG) values from mean fluorescence intensity (MFI) values and normalized to total β1 integrin expression (n = 8 mice per genotype). Error bars show standard deviations. (B) Western blot from HT-29 cells expressing a control siRNA or a siRNA directed against hKindlin-1 (siKind1) for Kindlin-1 and Kindlin-2. GAPDH was used to show equal loading. (C) Adhesion assay of control and Kindlin-1-depleted HT-29 cells on the indicated substrates (n = 5). Shown are mean values, error bars show standard errors of the mean. Coll IV, Collagen IV; FN, Fibronectin; LN332, Laminin-332; PLL, Poly-L lysine. (D) Kindlin-1 pull-down from IEC lysates using GST-tagged cytoplasmic β integrin tails. (E) Direct interaction of Kindlin-1 with β1 integrin cytoplasmic tails. His-tagged Kindlin-1 C-terminus was co-precipitated with GST-tagged β1 integrin cytoplasmic tails, but not with GST alone or an Y800A mutant form of β1 integrin. (F) Quantification of αIIbβ3 integrin activation, as measured by activation specific antibody PAC1 in CHO cells using flow cytometry (n = 9). Shown are mean values, error bars show standard deviations. (G) Pull-down with GST-tagged cytoplasmic β3 integrin tail from CHO cells transiently transfected with the indicated EGFP-constructs. (H) Western blot of CHO cells 24 hours after transfection with the indicated siRNAs. (siSCR: scrambled; siKind2_1 and siKind2_2: Kindlin-2 specific siRNAs) (I) Quantification of αIIbβ3 integrin activation in CHO cells transfected with the indicated cDNA constructs and/or siRNAs (n = 8). Shown are mean values, error bars show standard deviations.

An alternative explanation for the IEC detachment could be a reduction of integrin levels, or a dysfunction of integrins, similar to that reported for Kindlin-3-deficient platelets [8] and Kindlin-2-deficient primitive endoderm [18]. The normal distribution of β1 integrin (Figure 7B) and the comparable levels of β1 and αv (Itgav) integrins (Figure 7C and D and data not shown) excluded a defect in expression and/or translocation of integrins to the plasma membrane. Flow cytometry of primary IECs with the monoclonal antibody 9EG7, which recognizes an activation-associated epitope on the β1 integrin subunit, showed significantly reduced binding (Figure 8A) suggesting that loss of Kindlin-1 decreases activation (inside-out signaling) of β1 integrins. Primary keratinocytes from Kindlin-1^−/−^ mice also showed normal localization (Figure S10A) and surface expression of β1 integrins (Figure S10B). Interestingly, 9EG7 staining revealed reduced, although not statistically significant, activation of β1 integrins in these cells (Figure S10C).

Since it is difficult to culture and maintain primary murine IECs, we depleted Kindlin-1 in a human colon carcinoma cell line (HT-29) using RNAi (HT-29siKind1; Figure 8B) to show that integrin-mediated cell adhesion and shear stress induced detachment were also perturbed in a colon cell line. HT-29siKind1 cells were unable to adhere to Fibronectin (FN; Fn1) and showed strongly reduced adhesion to Laminin-332 and Collagen IV (Figure 8C) and easily detached from FN upon exposure to low (0,5dyn/cm^2^) as well as high shear stress (up to 4dyn/cm^2^; Figure S11). The remaining adhesion to Laminin-332 and Collagen IV is likely mediated by other Laminin- and Collagen-binding receptors on colonic epithelial cells such as α6β4 integrins (Itga6,Itgb4) and discoidin domain receptors [19],[20], which are both known to function independent of Laminin and Collagen binding β1 integrins [21].

These findings indicate that (i) loss of Kindlin-1 impairs integrin activation, which compromises adhesion of colonic epithelial cells, that (ii) Kindlin-2 cannot rescue Kindlin-1 loss in colonic epithelial cells, and that (iii) the residual IEC adhesion to Laminin and Collagen is suspended by shear forces exerted for example, by the feces.

It has been reported that Kindlin-1 associates with the membrane proximal NPxY motif of β1 and β3 integrins [6]. This observation, however, is in contrast with observations made with Kindlin-2 and -3, both of which bind the membrane distal NxxY motifs of β1 and β3 integrins to trigger their activation [8],[18],[22]. To explore the mechanism whereby Kindlin-1 induces integrin activation, we performed pull down experiments with recombinant GST-tagged cytoplasmic β integrin tails in IEC and keratinocyte lysates. The results confirmed that Kindlin-1 associated with the cytoplasmic domains of β1 and β3 (Figure 8D). Since substitutions of the tyrosine residues in the proximal NPxY motifs with alanine (β1Y788A; β3Y747A) allowed Kindlin-1 binding, while tyrosine to alanine substitutions in the distal NxxY motif of β1 and β3 integrin tails (β1Y800A; β3Y759A) abolished Kindlin-1 binding, we conclude that the binding and functional properties are conserved among all Kindlins. This was further confirmed with direct binding assays, which showed that the recombinant His-tagged C-terminal FERM domain of Kindlin-1 (aa 471–677) containing the phosphotyrosine binding (PTB) motif bound GST-tagged β1 but not the Y800A mutated β1 integrin cytoplasmic tail (Figure 8E).

It is well established that Talin can induce activation of integrins, and for a long time it was believed that it is sufficient for the execution of this task. This important function of Talin was discovered by overexpressing Talin or its FERM domain in CHO cells stably expressing the human platelet integrin αIIbβ3 (Itga2b, Itgb3) [23], which shifted the inactive conformation of αIIbβ3 integrin to a high affinity state, as demonstrated by increased binding of the PAC1 antibody recognizing activation associated epitopes on αIIbβ3 integrin (Figure 8F). In contrast to Talin, overexpression of Kindlin-1 failed to trigger activation of αIIbβ3 integrin in these cells (Figure 8F). Interestingly, as described for Kindlin-2 [18],[22], overexpression of both the Talin FERM domain and Kindlin-1 doubled PAC1 binding when compared with cells expressing only the Talin FERM domain (Figure 8F). The synergism between Talin and Kindlin-1 depends on a Kindlin-1 and β integrin tail interaction, as a PTB mutant of Kindlin-1 (QW611/612AA) failed to bind β integrin tails (Figure 8G) and the synergistic effect with Talin was lost (Figure 8F). These findings suggest that Kindlin-1 is not sufficient for integrin activation but is required for inducing Talin-mediated integrin activation. This notion was confirmed with CHO cells, in which endogenous Kindlin-2 levels were depleted by RNAi (Figure 8H). Furthermore, overexpressing Talin failed to induce integrin activation in these cells, but expression of Kindlin-1 restored this function (Figure 8I).

These findings show (i) that Kindlin-1 and -2 require Talin for integrin activation, (ii) that Talin requires Kindlins for integrin activation, and (iii) that Kindlin-1 and Kindlin-2 have redundant functions in vitro *as* both Kindlin-1 and -2 are recruited to FAs where they exert similar functions on integrin cytoplasmic tails. However, in vivo this is not the case as Kindlin-2 is recruited to cell-cell contacts in IECs and apparently does not compensate Kindlin-1 loss.

## Discussion

In the present study we show that a null mutation in the Fermt1 gene gives rise to skin atrophy and an acute and fulminant, neonatal intestinal epithelial dysfunction. We demonstrate that the primary defect is a loss of the intestinal epithelial barrier that secondarily leads to inflammatory cell infiltrates and the development of a severe colitis. Furthermore, we show that loss of the intestinal epithelial barrier is caused by a severe adhesion defect of intestinal epithelial cells to the underlying BM, which in turn is caused by the inability of integrins to become activated and to bind BM components. It is possible that in addition to defective integrin activation and epithelial detachment, Kindlin-1 exerts other yet unidentified functions that could contribute to the phenotype in Kindlin-1^−/−^ mice.

Kindler syndrome (KS) is thought to be primarily a skin disease with a disease course that is characterized by epidermal atrophy and followed by epidermal blistering, pigmentation defects and skin cancer. The complex disease syndrome is difficult to diagnose at the disease onset due to similarities with other forms of skin blistering diseases (also called epidermolysis bullosa; EB) that are caused by mutations in keratin and BM genes [24]. Recent case reports showed that KS may involve more organs than only the skin, as several KS patients also suffer from intestinal symptoms. One patient with a severe form of KS developed a severe postnatal UC. Interestingly, this patient was diagnosed with a null mutation in the FERMT1 gene after developing trauma-induced skin blistering [10]. In line with this severe UC case of KS, we found that the null mutation of the Fermt1 gene in mice also leads to a dramatic and lethal intestinal epithelial dysfunction very shortly after birth. Lethality is usually not seen in KS patients, which is most likely due to the immaturity of the murine intestine at birth, making it more vulnerable to injury [25].

The intestinal epithelial dysfunction of Kindlin-1-deficient mice is characterized by flat and superficial ulcerations in the colon, as the epithelium detaches from an intact BM. The defects begin in the rectum and extend along the entire colon finally leading to a severe pancolitis. The ulcerations and epithelial cell detachments are restricted to the colon, although the ileum shows signs of a secondary inflammation at later stages of the disease. In vitro studies with primary IECs from Kindlin-1^−/−^ mice and Kindlin-1-depleted HT-29 cells showed that the cell detachment is caused by impaired activation of integrins leading to weak adhesion of IECs to the underlying BM. Mechanistically Kindlin-1 requires direct binding to the β1 and β3 integrin cytoplasmic domains to promote the activation of the two integrin subfamilies. These biochemical and functional properties are conserved among the three members of the Kindlin family. Kindlin-2-mediated binding and activation of β1 and β3 integrins critically support the attachment of endoderm and epiblast cells to the underlying BM in peri-implantation embryos [18], while Kindlin-3 plays a central role for the activation of platelet integrins [8]. The findings of this report also demonstrate that Talin function crucially depends on the activity of Kindlin-1. Depletion of Kindlin-2 (the only Kindlin expressed in CHO cells) completely prevents overexpressed Talin from activating integrins. Re-expression of either Kindlin in Kindlin-2-depleted CHO cells, however, recovers the ability of Talin to trigger the activation of integrins. It will be important to next investigate how Kindlins become activated and why Kindlin-2 is unable to take over the function of Kindlin-1 in Kindler Syndrome and Kindlin-1-deficient mice.

The conclusion that the observed phenotype is triggered by IEC detachment rather than by a primary inflammatory defect in Kindlin-1 deficient mice is based on the observation that epithelial cell detachment always occurred prior to immune cell infiltration. We would therefore, argue that the detachment of IECs resembles an intestinal wound, which secondarily triggers a strong wound healing response leading to immune cell infiltrates and release of a cytokine storm. In line with this hypothesis, epithelial cell detachment and induction of inflammatory reactions can be completely prevented when Kindlin-1 pups are delivered by Caesarian section and subsequently incubated in a humidified and temperature controlled chamber. Mechanical stress applied by the colostrum is likely inducing the detachment of the weakly adhering epithelial cells in the colon. The vast majority of mouse models reported to develop colitis so far have an abnormal immune system [12],[26]. This fact as well as the identification of several susceptibility loci in human patients [14],[15] led to the conclusion that defects in the immune system are of central importance for UC development. Severe adhesion defects of IECs leading to a massive wound response may represent an alternative etiology for UC development.

Although adhesion is severely compromised in the colon of Kindlin-1-deficient mice, they are born without skin blisters. This is in line with KS patients, who are also born without skin blisters even when they are delivered by the vaginal route but develop blisters postnatally at trauma prone sites. Interestingly, Kindlin-1 deficient mice did not show defective adhesion of basal keratinocytes to the BM even after application of mechanical stress. The different severity of the adhesion defect in skin and colon could be reflected by the functional properties of the distinct set of integrins expressed in the two organs and the absence of classical hemidesmosomes in intestinal epithelial cells [27].

Another pronounced skin defect in Kindlin-1-deficient mice as well as KS patients is skin atrophy, which seems to be due to reduced proliferation of interfollicular keratinocytes. This finding raises several questions; first, regarding the mechanism underlying the molecular control of cell proliferation by Kindlin-1. The mechanism is unknown and could result from a diminished cross talk between integrin and growth factor signaling. Second, it is also unclear how a molecular player that supports proliferation is giving rise to cancer at a later stage. It is possible that the localization of Kindlins in different cellular compartments, i.e. cell-matrix adhesion sites, cell-cell adhesion sites and in certain instances in the nucleus, equips them with different functions that become evident at different time points in life.

Kindlin-1 and -2 are co-expressed in epidermal cells as well as epithelial cells of the colon. Interestingly, we found that Kindlin-2 cannot compensate Kindlin-1 function in vivo, neither in the colon nor in skin. Since Kindlin-2 normally localizes to cell-cell adhesions in both cell types and does not translocate to integrin adhesion sites in mutant intestinal and epidermal epithelial cells, it is unable to compensate for the loss of Kindlin-1, although both Kindlins are capable of performing the same tasks at the integrin tails *ex vivo* and in vitro [27]. Hence, a therapeutic strategy to reroute some of the Kindlin-2 from cell-cell to the integrin adhesion sites may represent a promising approach to prevent ulceration in KS patients with severe UC.

## Materials and Methods

### Mouse Strains

The Kindlin-1^−/−^ mice were obtained by replacing the ATG-containing exon 2 with a neomycin resistance cassette (detailed information on the cloning of the targeting construct can be obtained from Faessler@biochem.mpg.de). The construct was electroporated into R1 embryonic stem (ES) cells (passage 15) and homologous recombination was verified with southern blots. Genomic DNA was digested with EcoRV, blotted and then hybridized with a 5′ probe or digested with BglII, blotted and hybridized with a 3′ probe (Figure 1A). Targeted ES cells were injected into blastocysts and transferred into foster mice. Mice were housed in a special pathogen free mouse facility. All animal experiments have been approved by the local authorities.

### Histology, Immunohistochemistry, and Immunfluorescence Stainings

For H&E stainings intestinal segments were either PFA fixed and embedded in paraffin, or frozen on dry-ice in cryo-matrix (Thermo). Immunhistochemistry of paraffin embedded sections was carried out as previously described [5]. Sections of 8 µm thickness were prepared and stained following routine protocols. Cryo sections were fixed in 4% PFA/PBS except for the Kindlin-1 staining where sections were fixed with 1∶1 methanol/acetone at −20°C. Subsequently tissue sections were blocked with 3% BSA/PBS, incubated with primary antibodies in a humidity chamber over night at 4°C, with fluorescently labeled secondary antibodies for 1 h at RT and finally mounted in Elvanol. Pictures were taken with a Leica DMIRE2 confocal microscope with a 100× or 63× NA 1.4 oil objective.

### GST-Pull Downs

Recombinant GST-β1, β1Y788A, β1Y800A, β3, β3Y747A, β3Y759A cytoplasmic tails were expressed and purified from *E.coli* under non denaturing conditions. 5 µg of recombinant tails were incubated with 500 µg IEC lysate (in 50 mM Tris pH 7.4, 150 mM NaCl, 1 mM EDTA, 1% Triton-X-100) overnight. GST-constructs were precipitated with glutathione beads (Novagen). Subsequent western blots were probed for Kindlin-1 and GST.

### Antibodies

A polyclonal peptide antibody against Kindlin-1 was raised against the peptide YFKNKELEQGEPIEK as previously described [5].

The following antibodies were used at the given concentration indicated for western blot (W), immunoprecipitation (IP), immunfluorescence (IF), immunhistochemistry (IHC): Kindlin-1 (W: 1∶5000, IF cells: 1∶200, IF tissue: 1∶1000), Kindlin-2 (W: 1∶1000, IF cells 1∶200, IF tissue: 1∶200), E-cadherin (Cdh1; Zymed, W: 1∶5000), Migfilin (W: 1∶5000, IF cells 1∶100, IF tissue: 1∶100), GAPDH (Chemicon; W: 1∶10000), phalloidin Tritc (Sigma; IF cells: 1∶800, IF tissue: 1∶800), Mac-1 (EuroBioscience; IF tissue: 1∶100), GR-1 (Ly6g; eBioscience; IF tissue: 1∶100), Thy1.2 (PharMingen; IF tissue: 1∶100), GST (Novagen; W: 1∶10000), His (CellSignaling; W: 1∶1000), PAC1 (BD; FACS: 1∶100), α6 integrin (Itga6; PharMingen; IF tissue: 1∶100), CollagenIV (a gift from Dr. Rupert Timpl; IF tissue: 1∶100), Laminin-332 (a gift from Dr. Monique Aumeilley; IF tissue: 1∶200), Perlecan (Hspq2; a gift from Dr. Rupert Timpl; IF tissue: 1∶100), β1 integrin (Chemicon; WB: 1∶3000, IF tissue: 1∶600), 9EG7 (PharMingen; FACS: 1∶100), EGFP (Abcam; WB: 1∶10000), β1 integrin (PharMingen; FACS: 1∶200), αv integrin (PharMingen; FACS: 1∶200). Keratin10 (Krt10; Covance; IHC: 1∶600 ), Keratin14 (Krt14; Covance; IHC: 1∶600 ), Loricrin (Lor; Covance; IHC: 1∶500 ) Ki67 (Dianova; IHC: 1∶50), cCaspase3 (CellSignaling, IHC: 1∶100).

### Caesarean Section

Pregnant mice were sacrificed by cervical dislocation when embryos were at E18.5–E19 of gestation. The uterus was removed and cut open. Embryos were taken out and the umbilical cord was cut. Mice were subsequently dried and kept in an incubator at 37°C and high humidity.

### Real Time PCR

Total RNA from whole colons was extracted with a PureLink Micro to Midi RNA extraction kit (Invitrogen) following the manufacturers instructions. cDNA was prepared using the iScript cDNA Synthesis Kit (Biorad). Real Time PCR using a Sybr Green ready mix (Biorad) was performed in an iCycler (Biorad). Each sample was measured in triplicates and values were normalized to GAPDH. Following primers were used; TNFα fwd: AAAATTCGAGTGACAAGCCTGTAGC, TNFα rev: GTGGGTGAGGAGCACGTAG. IL-6 fwd: CTATACCACTTCACAAGTCGGAGG IL-6 rev: TGCACAACTCTTTTCTCATTTCC. RT-PCR for Kindlin-1 and GAPDH was performed as previously described [5].

### Isolation of IECs

Neonatal mouse intestine was removed and flushed with 1 ml PBS. The intestine was longitudinally cut open, rinsed with PBS and incubated for 40 min. in IEC isolation buffer (130 mM NaCl, 10 mM EDTA, 10 mM Hepes pH 7.4, 10% FCS and 1 mM DTT) at 37°C on a rotor. The epithelium was shaken off and pelleted by centrifugation at 2000rpm for 5 min. For WB analysis cells were washed once with PBS and subsequently lysed. For flow cytometry cells were washed once with PBS and trypsinized with 2× trypsin (GIBCO) for 10 min. at 37°C. Trypsin was inactivated with DMEM containing 10%FCS. A single cell suspension was prepared by passing cells through a cell strainer (BD).

### Isolation of Keratinocytes

Primary keratinocytes were isolated from P3 mice as described previously [28]. Cells were cultured on a mixture of ColI (Cohesion) and 10 µg/ml FN (Invitrogen) coated plastic dishes in keratinocyte growth medium containing 8% chelated FCS (Invitrogen) and 45 µM Ca^2+^.

### Flow Cytometry

IEC's and keratinocytes were stained with 9EG7 antibody in Tris buffered saline [29]. For the PAC1 binding assay CHO cells were stained for 40 min. at RT as described previously [23]. Cells were gated for viability by excluding propidium iodide-positive cells. CHO cells transfected with EGFP-tagged constructs were additionally gated for highly EGFP-positive cells. Measurements were performed with a FACS Calibur (BD) and data evaluation was done with FlowJo software.

### Constructs

The EGFP-Kindlin-1 expression plasmid was previously described [5]. The cDNA encoding His-Kindin-1 C-terminus (aa 471–677) was amplified by PCR and cloned into the pQE-70 vector (Qiagen). The Kindlin-1 PTB mutation QW611/612AA was introduced with a site directed mutagenesis kit (Stratagene) following the manufacturers recommendations. All EGFP constructs were cloned into the pEGFP-C1 vector (Clontech) and subsequently sequenced. EGFP-Talin head was previously described [8].

### Cell Culture

CHO and HT-29 cells were maintained in DMEM containing penicillin/streptomycin, non-essential amino-acids and 10% or 20% FCS, respectively (GIBCO). Cells were transfected with 2 µg of each DNA in six well plates using Lipofectamine 2000 following the manufacturers' instructions (Invitrogen).

### RNAi

To deplete Kindlin-1 constitutively from HT-29 cells, an shDNA corresponding to the cDNA sequence GTAAGTCCTGGTTTATACA of hKindlin-1 and a control cDNA with the sequence AGCAGTGCATGTATGCTTC were cloned into the pSuperRetro vector (OligoEngine). Viral particles were prepared as described previously [30]. HT-29 cells were infected and subsequently selected with 2mg/l puromycin. Transient knockdown of Kindlin-2 from CHO cells was achieved by transfection of the siRNAs; Kind2_1: GCCUCAAGCUCUUCUUGAUdTdT and Kind2_2: CUCUGGACGGGAUAAGGAUdTdT, and a control siRNA (purchased from Sigma) using Lipofectamine 2000 (Invitrogen), following the manufacturers instructions. Cells were harvested and assayed 24 hours after transfection.

### Adhesion Assay

The adhesion assays were performed as previously described [29], using 40000 cells per well in serum free DMEM (HT-29) or MEM (primary keratinocytes).

### Osmolarity

Osmolarity was measured from 50 µl urine using an Osmomat 030 from Gonotec.

### X-Gal Barrier Assay

Embryonic skin barrier formation was determined as previously described [31].

### Shear Stress Assay

Slides with a 1 µm diameter (ibidi BioDiagnostics) were coated overnight with 5 µg/ml FN and then blocked with 1% BSA. 100.000 cells were seeded onto the slides and incubated for 2.5 hours in a cell culture incubator. Cells were subsequently exposed to increasing amounts of shear force in two minute intervals (as indicated in the Figure S11) and pictures were taken every second.

### Co-Immunoprecipitation

CHO cells were transiently transfected with the indicated EGFP constructs. Approximately 1mg of protein lysate was immunoprecipitated using the μMACS Epitope Tag Protein Isolation Kit for EGFP tags (Miltenyi Biotec) following the manufacturers instructions.

### Electron Microscopy

Electron microscopy was performed as previously described [29].

### Statistical Analysis

Analyses were performed with GraphPad Prism. If not mentioned otherwise in the figure legends, Gaussian distribution of datasets was determined by a D'Agostino & Pearson omnibus normality test. If samples were not Gaussian distributed a Mann-Whitney test was performed. Gaussian distributed samples were either compared with a one way ANOVA and a Tukey's multiple comparison post test or an unpaired two-tailed t-test.

## Supporting Information

Figure S1 Normal triglyceride and glucose levels in the blood of P3 Kindlin-1^−/−^ mice. Triglyceride (n = 7 per genotype) and glucose (n = 5 per genotype) levels from total blood at P3. The differences are statistically insignificant. Shown are mean values, error bars show standard deviations.
(0.5 MB TIF)

Figure S2 Normal kidney morphology in Kindlin-1^−/−^ mice. (A) Coomassie stained gel of 10 µl urine from P2 and P3 control and Kindlin-1^−/−^ mice. (B) Quantification of total protein in urine from Kindlin-1^+/+^, Kindlin-1^+/−^ and Kindlin-1^−/−^ mice at P1 (n = 14/7/6) and P3 (n = 20/20/9). Error bars show standard deviation. (C) H&E staining of P3 Kindlin-1^+/+^ and Kindlin-1^−/−^ kidneys. Kindlin-1^−/−^ kidneys do not show altered glomeruli and collecting duct morphology. Scale bar represents 200 µm. Enlargements show glomeruli (A1, A3) and collecting ducts (A2, A4). Scale bar represents 50 µm.
(8.6 MB TIF)

Figure S3 Normal BM composition and deposition in P3 Kindlin-1^−/−^ backskin. P3 backskin of wild type and Kindlin-1^−/−^ mice was stained for the BM components Laminin-332 (LN332; red), Collagen IV (Coll IV; red) and Fibronectin (FN; red) and co-stained with α6 or β4 integrin marking (green) the basal site of basal keratinocytes. The stainings reveal no differences in BM deposition and composition or α6 and or β4 integrin localization between control and Kindlin-1^−/−^ littermates. Scale bar indicates 30 µm.
(9.8 MB TIF)

Figure S4 Normal skin differentiation. P3 backskin of control and Kindlin-1^−/−^ mice was stained for epidermal differentiation markers Keratin14, Keratin10 and Loricrin (red) and co-stained with α6 integrin (green) to mark basal keratinocytes. The stainings show no difference in the differentiation pattern of Kindlin-1^−/−^ keratinocytes. Scale bar indicates 10 µm.
(8.4 MB TIF)

Figure S5 Normal skin development. (A) Normal X-Gal staining in E17 Kindlin-1^−/−^ embryos indicating normal barrier formation during development. Scale bar indicates 5 mm. (B) H&E staining of back skin from control and Kindlin-1^−/−^ littermates of different age. In Kindlin-1^−/−^ mice the epidermal (e) thickness at E18.5 and P0 is normal but clear epidermal atrophy is seen at P1. Scale bar indicates 50 µm. (d): dermis.
(8.5 MB TIF)

Figure S6 Altered adhesion and spreading of Kindlin-1^−/−^ keratinocytes. (A) Adhesion assay of control and Kindlin-1^−/−^ keratinocytes on LN332, Laminin-332; Coll IV, Collagen IV; FN, Fibronectin; PLL, Poly-L lysine (n = 3). Shown are mean values, error bars show standard error of the mean. (B) Cell area measured upon spreading on 5 µg/ml Fibronectin at the indicated time-points using MetaMorph software (n = 30 cells per genotype from 3 independent experiments). Shown are mean values, error bars show standard deviation (*** p<0.0001).
(0.8 MB TIF)

Figure S7 Oral and oesophageal mucosa in Kindlin-1^−/−^ mice. Histology of the oral mucosa and the oesophagus of P3 Kindlin-1^+/+^ and Kindlin-1^−/−^ mice did not reveal an abnormal morphology. Scale bar represents 50 µm.
(8.1 MB TIF)

Figure S8 Progressive epithelial loss in Kindlin-1^−/−^ colons. (A) Quantification of the extent of intact colonic epithelium at P1 and P3 (n = 3 per genotype and age). Error bars show range. (B) Overview of an H&E picture of a P3 control and Kindlin-1^−/−^ colon. The Kindlin-1^−/−^ colon shows a complete absence of colonic epithelium (black arrowheads), while the epithelium in the caecum is still present (white arrowheads). Scale bars show 500 µm. (C) Magnifications of the boxed areas shown in B.
(7.7 MB TIF)

Figure S9 Normal IEC proliferation but detachment induced apoptosis . P1 colons from wild type and Kindlin-1^−/−^ mice were DAB stained for cleaved Caspase-3 to determine apoptosis and Ki67 stained to show proliferating IECs. Apoptosis occurs in detached epithelium of Kindlin-1^−/−^ mice. In areas of still adhering epithelium the number of proliferating IECs is similar between wild type and Kindlin-1^−/−^ mice. Scale bar indicates 50 µm.
(8.7 MB TIF)

Figure S10 β1 integrin activation in Kindlin-1^−/−^ keratinocytes. (A) Immunofluorescence staining for β1 integrin (green) and Laminin-332 (LN332; red) from P3 backskin shows normal localization of β1 integrin in Kindlin-1^−/−^ backskin. (B) FACS quantification of β1 integrin expression of freshly isolated control and Kindlin-1^−/−^ keratinocytes at P2 shows unaltered β1 integrin expression on basal keratinocytes. Error bars show range (n = 4) (C) 9EG7 FACS quantification of these keratinocytes shows no significant reduction in β1 integrin activation. Error bars show range (n = 4).
(8.0 MB TIF)

Figure S11 Shear induced detachment of Kindlin-1 depleted HT-29 cells. Control and Kindlin-1 depleted HT-29 (siKind1) cells were plated on Fibronectin-coated flow chamber slides and exposed to increasing shear forces as indicated in the figure. Control cells did not detach from the matrix while Kindlin-1 depleted cells were unable to resist low or high shear forces (compare lane 1 (0dyn/cm^2^) with lane 2 (0,5dyn/cm^2^). Scale bar indicates 50 µm.
(9.0 MB TIF)
