# Supplementary figures and images for: Loss of Kindlin-1 Causes Skin Atrophy and Lethal Neonatal Intestinal Epithelial Dysfunction
Source: PLoS Genet. 2008 Dec 5;4(12):e1000289. doi: 10.1371/journal.pgen.1000289 (PMC2585060; doi:10.1371/journal.pgen.1000289)

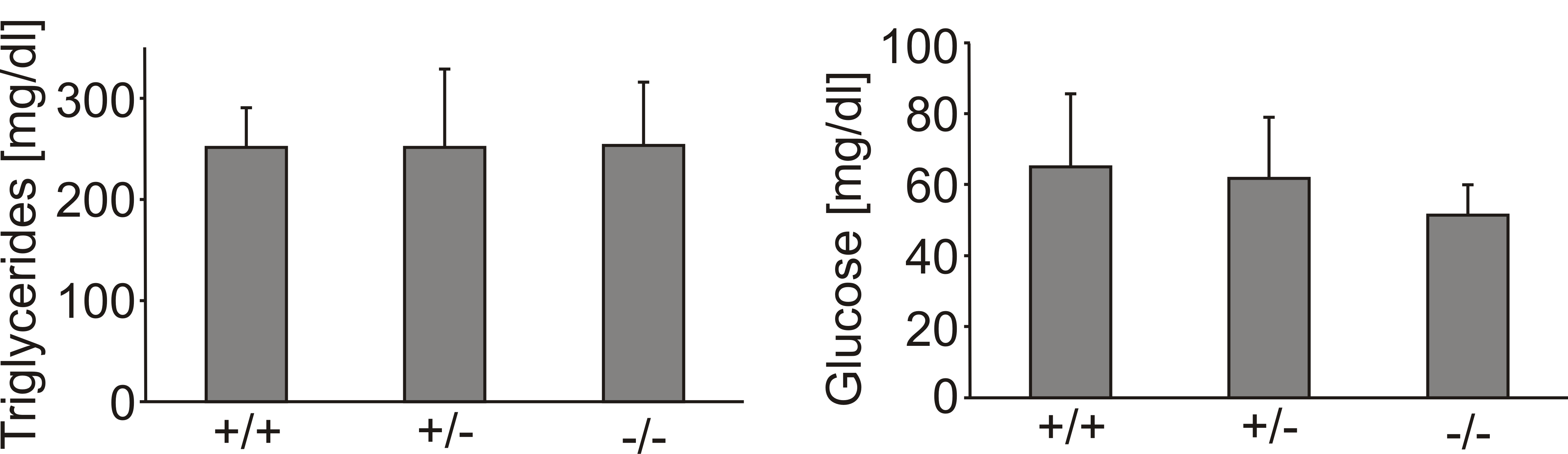

Supplement: Figure S1 — Normal triglyceride and glucose levels in the blood of P3 Kindlin-1−/− mice. Triglyceride (n = 7 per genotype) and glucose (n = 5 per genotype) levels from total blood at P3. The differences are statistically insignificant. Shown are mean values, error bars show standard deviations. (0.5 MB TIF) [file pgen.1000289.s001.tif]

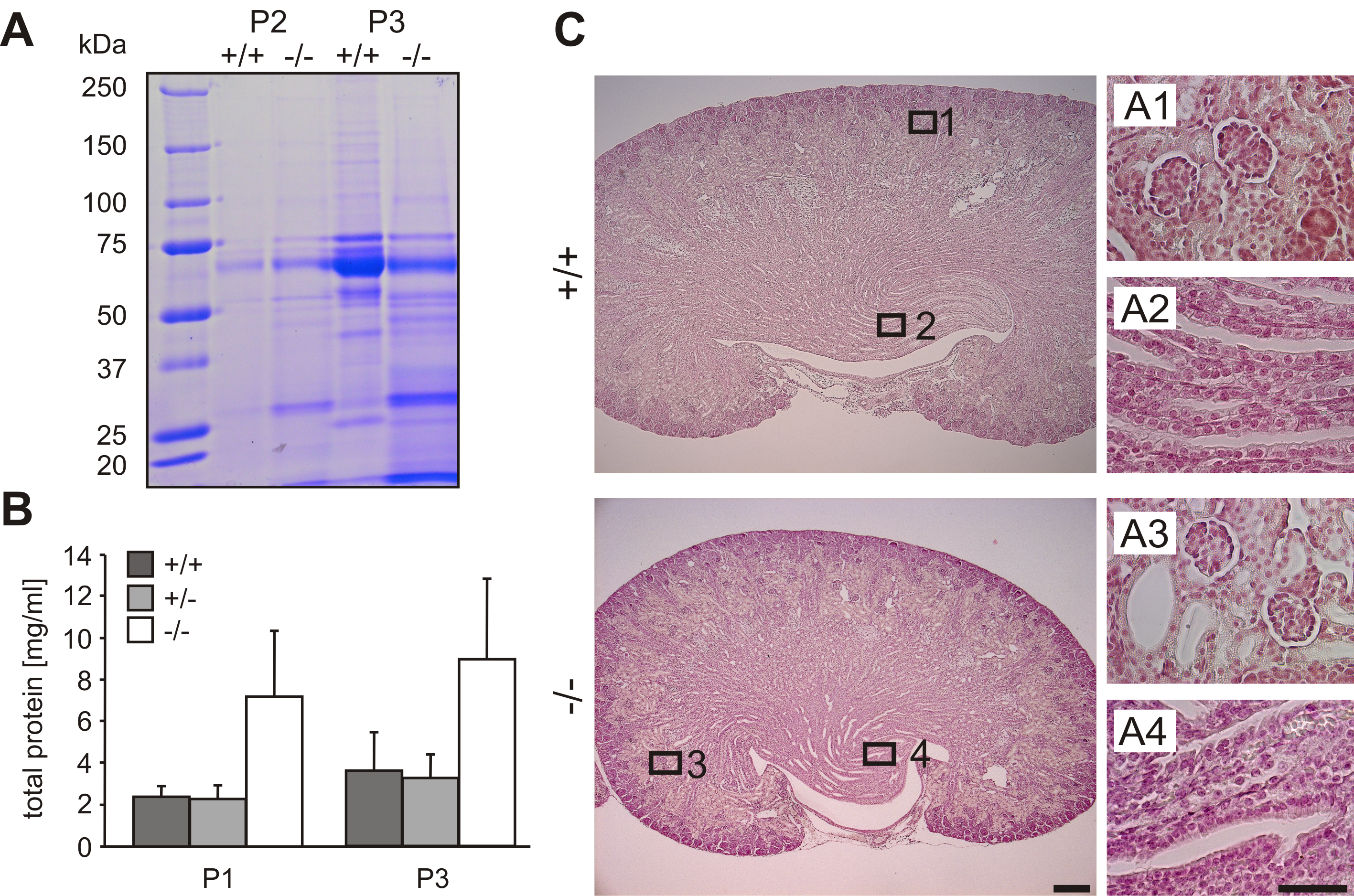

Supplement: Figure S2 — Normal kidney morphology in Kindlin-1−/− mice. (A) Coomassie stained gel of 10 µl urine from P2 and P3 control and Kindlin-1−/− mice. (B) Quantification of total protein in urine from Kindlin-1+/+, Kindlin-1+/− and Kindlin-1−/− mice at P1 (n = 14/7/6) and P3 (n = 20/20/9). Error bars show standard deviation. (C) H&E staining of P3 Kindlin-1+/+ and Kindlin-1−/− kidneys. Kindlin-1−/− kidneys do not show altered glomeruli and collecting duct morphology. Scale bar represents 200 µm. Enlargements show glomeruli (A1, A3) and collecting ducts (A2, A4). Scale bar represents 50 µm. (8.6 MB TIF) [file pgen.1000289.s002.tif]

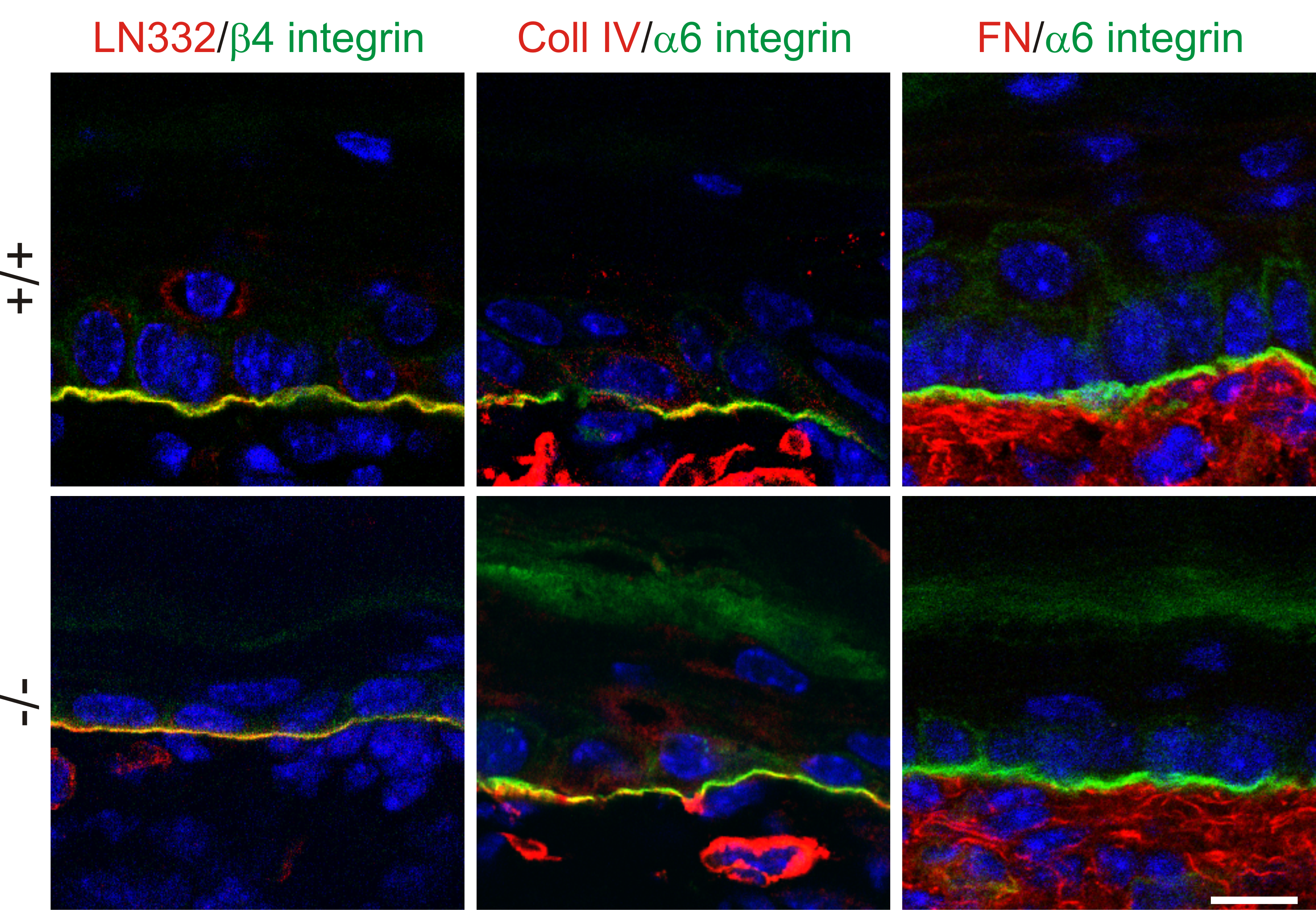

Supplement: Figure S3 — Normal BM composition and deposition in P3 Kindlin-1−/− backskin. P3 backskin of wild type and Kindlin-1−/− mice was stained for the BM components Laminin-332 (LN332; red), Collagen IV (Coll IV; red) and Fibronectin (FN; red) and co-stained with α6 or β4 integrin marking (green) the basal site of basal keratinocytes. The stainings reveal no differences in BM deposition and composition or α6 and or β4 integrin localization between control and Kindlin-1−/− littermates. Scale bar indicates 30 µm. (9.8 MB TIF) [file pgen.1000289.s003.tif]

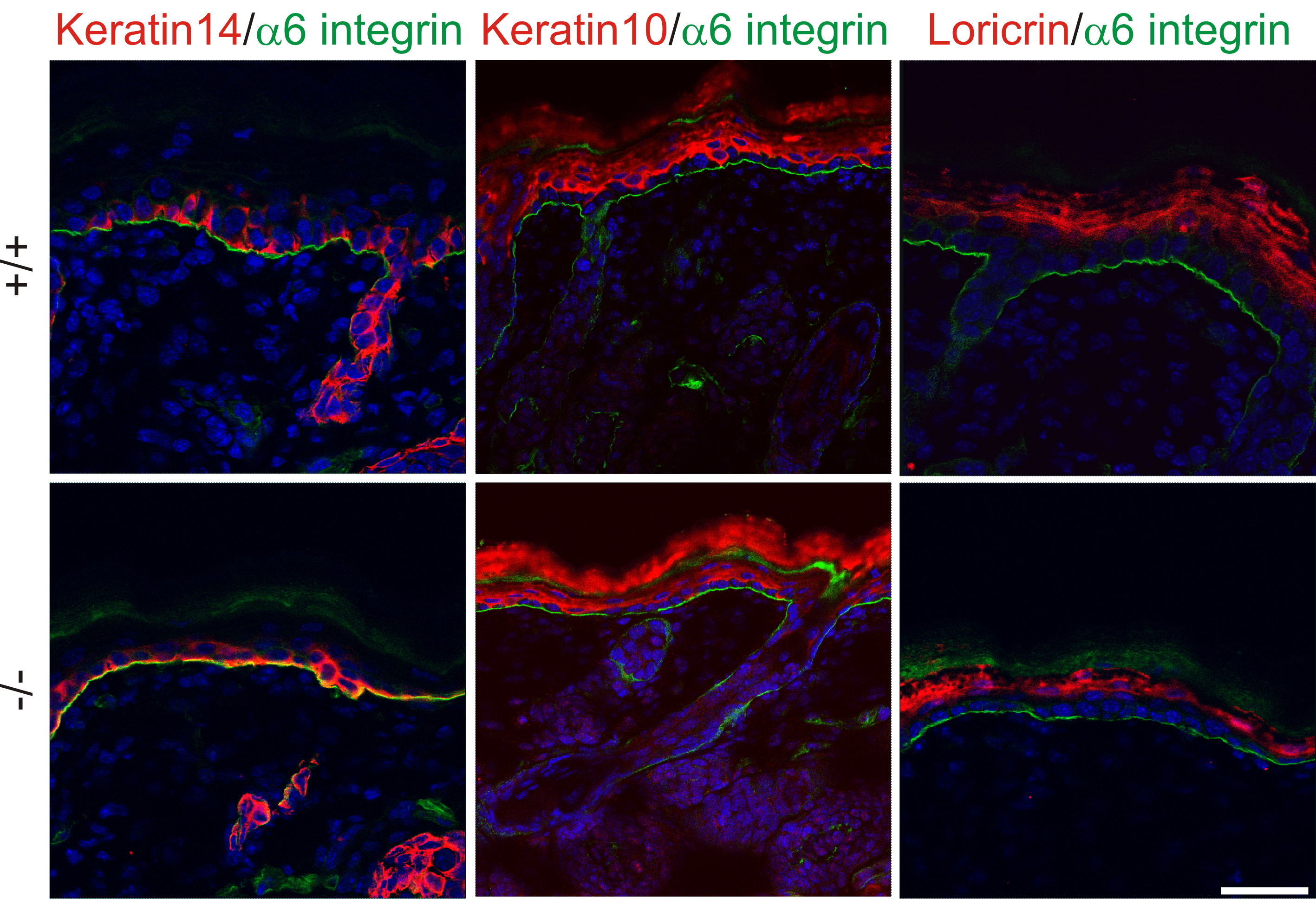

Supplement: Figure S4 — Normal skin differentiation. P3 backskin of control and Kindlin-1−/− mice was stained for epidermal differentiation markers Keratin14, Keratin10 and Loricrin (red) and co-stained with α6 integrin (green) to mark basal keratinocytes. The stainings show no difference in the differentiation pattern of Kindlin-1−/− keratinocytes. Scale bar indicates 10 µm. (8.4 MB TIF) [file pgen.1000289.s004.tif]

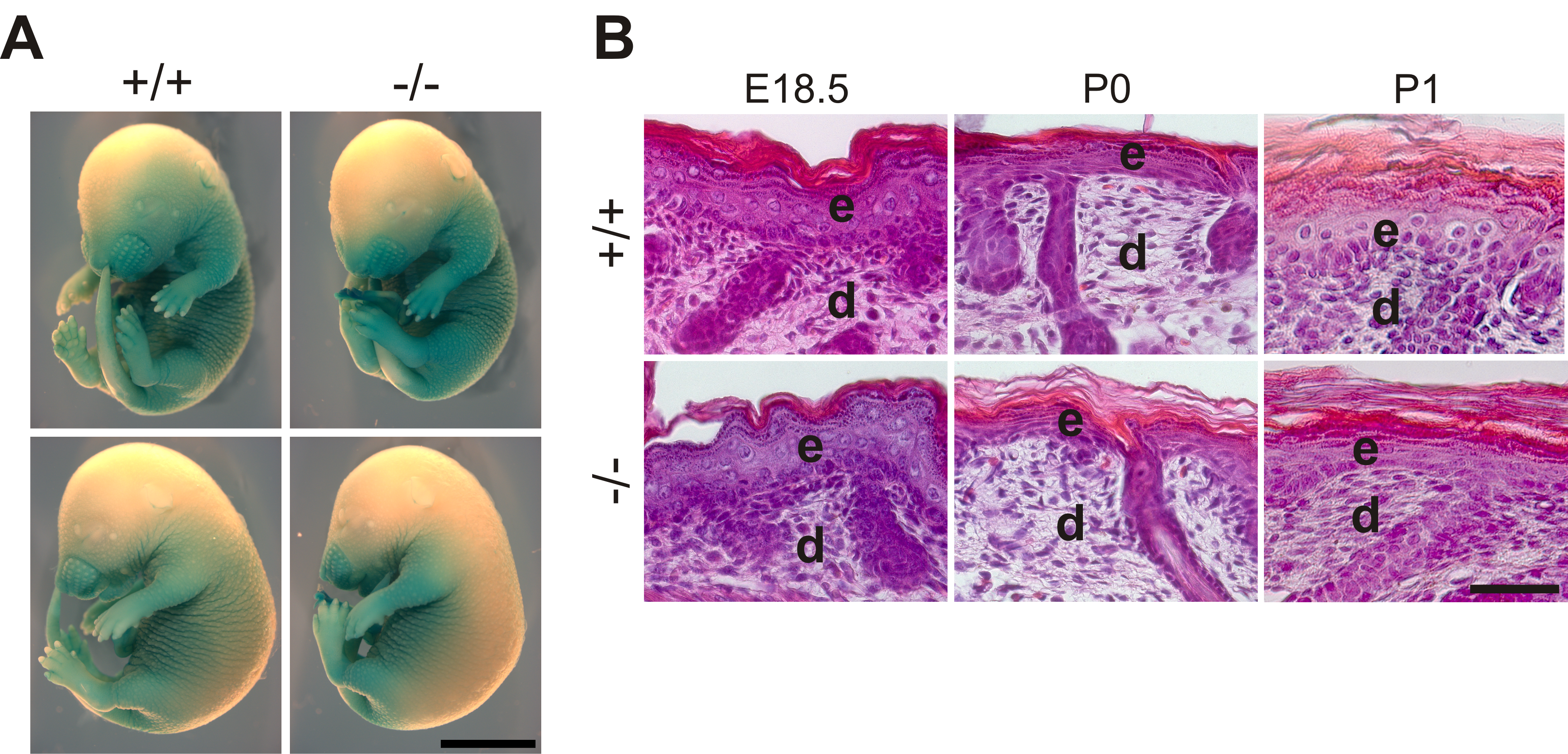

Supplement: Figure S5 — Normal skin development. (A) Normal X-Gal staining in E17 Kindlin-1−/− embryos indicating normal barrier formation during development. Scale bar indicates 5 mm. (B) H&E staining of back skin from control and Kindlin-1−/− littermates of different age. In Kindlin-1−/− mice the epidermal (e) thickness at E18.5 and P0 is normal but clear epidermal atrophy is seen at P1. Scale bar indicates 50 µm. (d): dermis. (8.5 MB TIF) [file pgen.1000289.s005.tif]

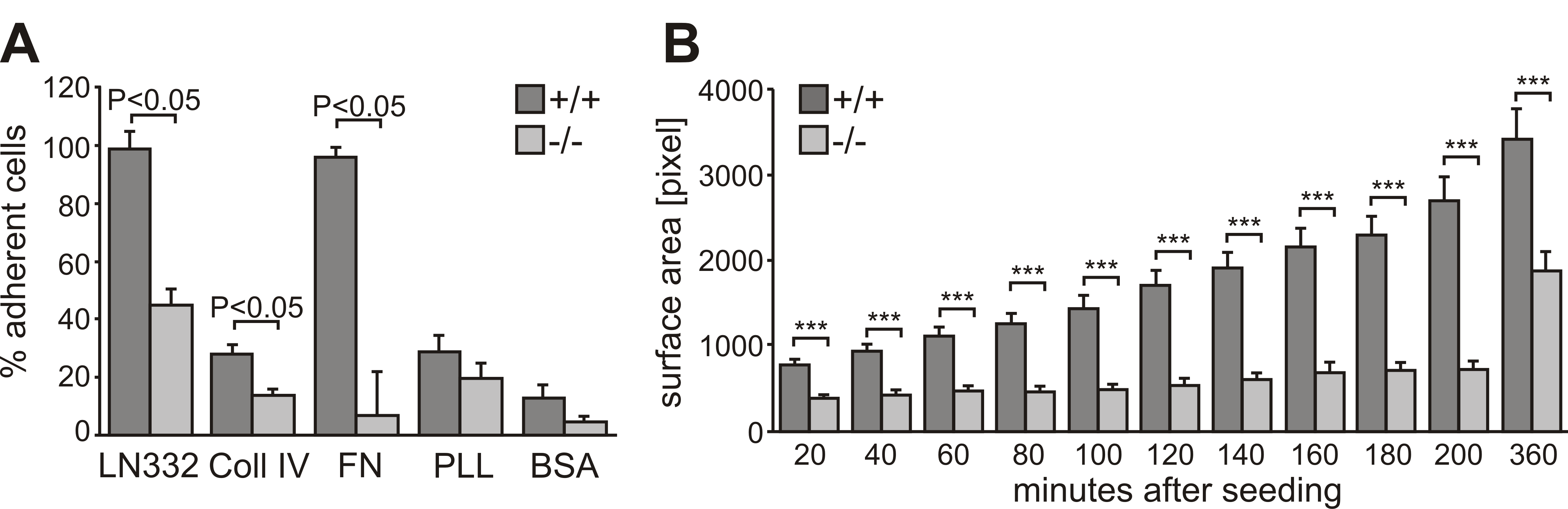

Supplement: Figure S6 — Altered adhesion and spreading of Kindlin-1−/− keratinocytes. (A) Adhesion assay of control and Kindlin-1−/− keratinocytes on LN332, Laminin-332; Coll IV, Collagen IV; FN, Fibronectin; PLL, Poly-L lysine (n = 3). Shown are mean values, error bars show standard error of the mean. (B) Cell area measured upon spreading on 5 µg/ml Fibronectin at the indicated time-points using MetaMorph software (n = 30 cells per genotype from 3 independent experiments). Shown are mean values, error bars show standard deviation (*** p<0.0001). (0.8 MB TIF) [file pgen.1000289.s006.tif]

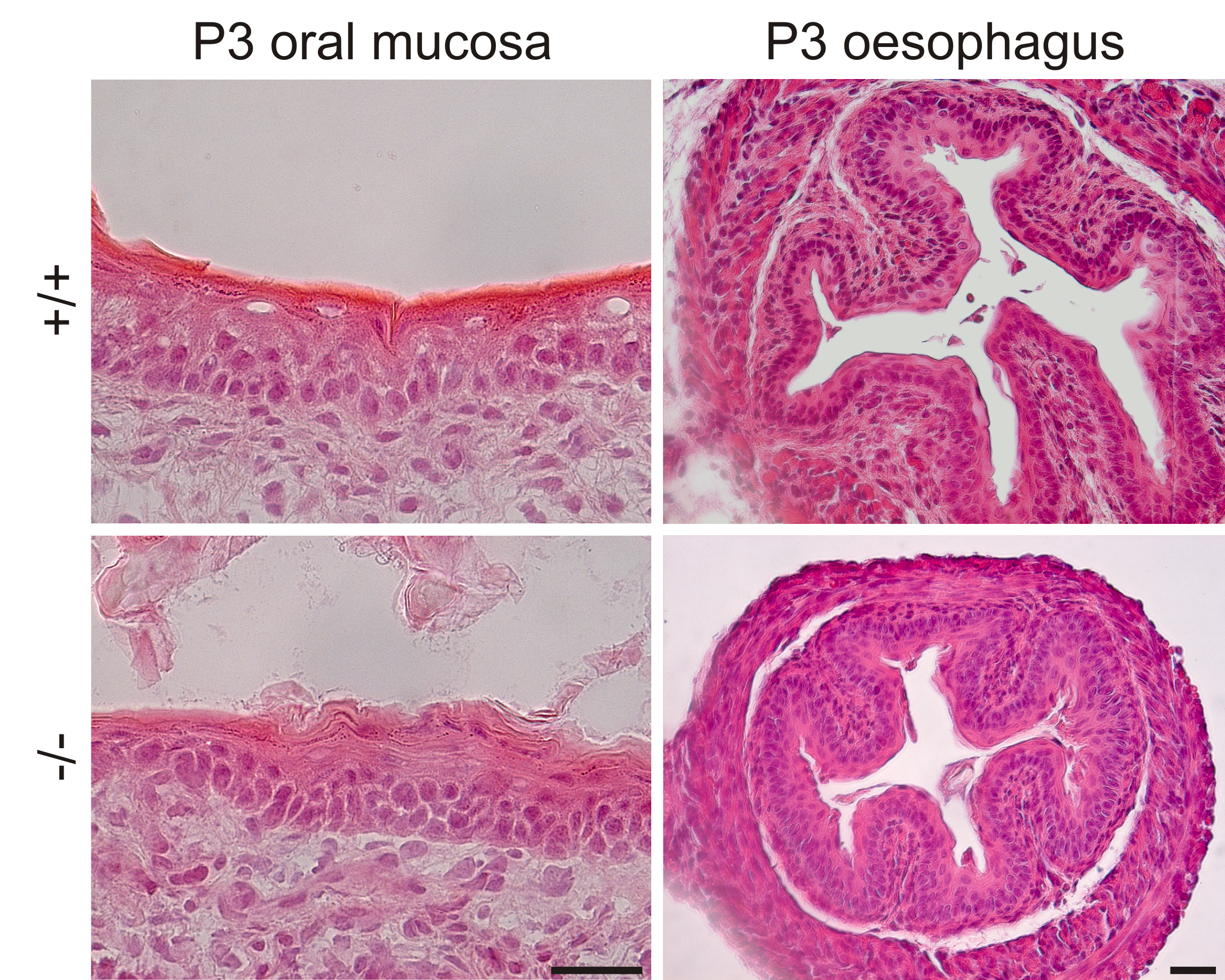

Supplement: Figure S7 — Oral and oesophageal mucosa in Kindlin-1−/− mice. Histology of the oral mucosa and the oesophagus of P3 Kindlin-1+/+ and Kindlin-1−/− mice did not reveal an abnormal morphology. Scale bar represents 50 µm. (8.1 MB TIF) [file pgen.1000289.s007.tif]

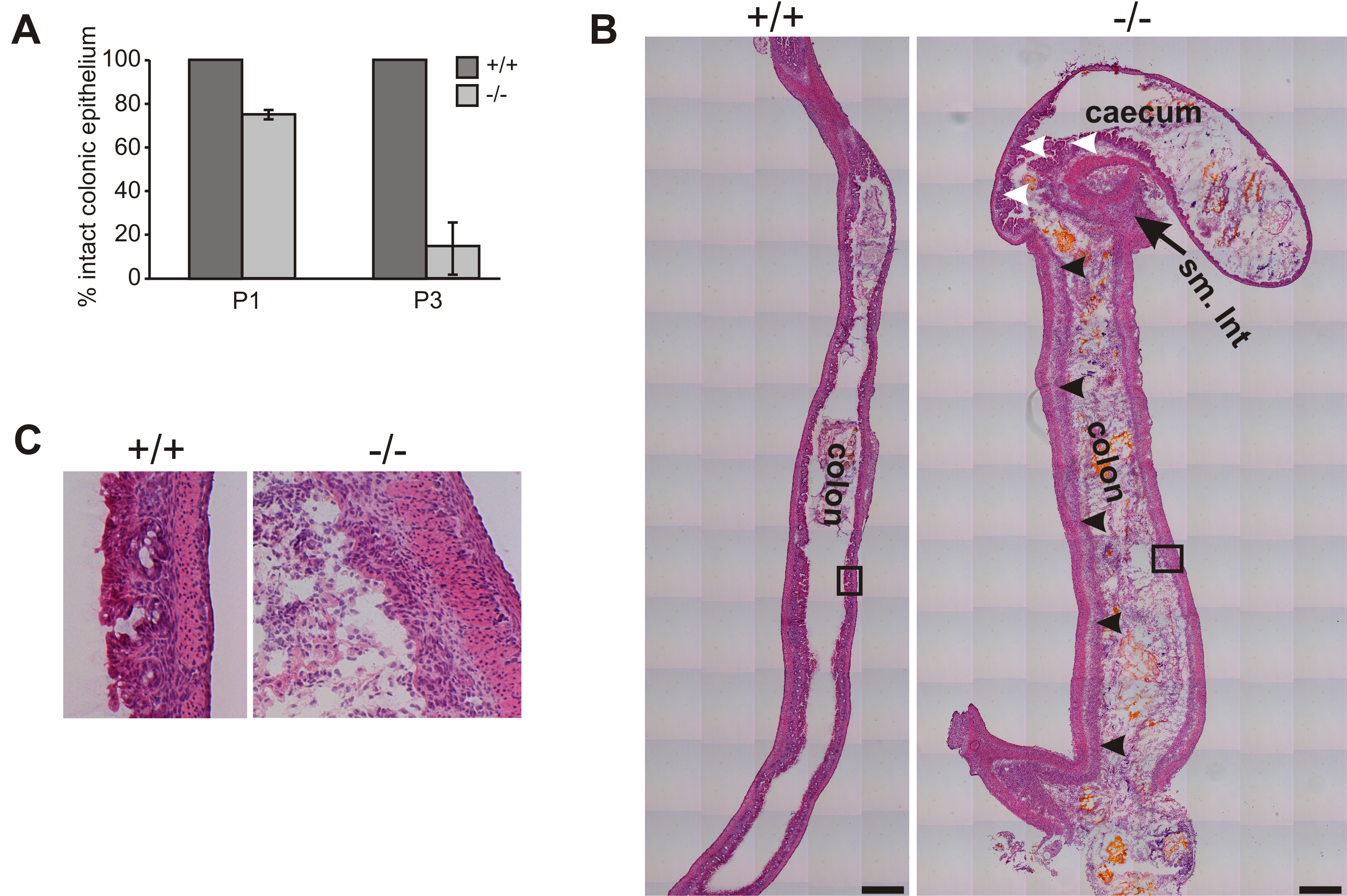

Supplement: Figure S8 — Progressive epithelial loss in Kindlin-1−/− colons. (A) Quantification of the extent of intact colonic epithelium at P1 and P3 (n = 3 per genotype and age). Error bars show range. (B) Overview of an H&E picture of a P3 control and Kindlin-1−/− colon. The Kindlin-1−/− colon shows a complete absence of colonic epithelium (black arrowheads), while the epithelium in the caecum is still present (white arrowheads). Scale bars show 500 µm. (C) Magnifications of the boxed areas shown in B. (7.7 MB TIF) [file pgen.1000289.s008.tif]

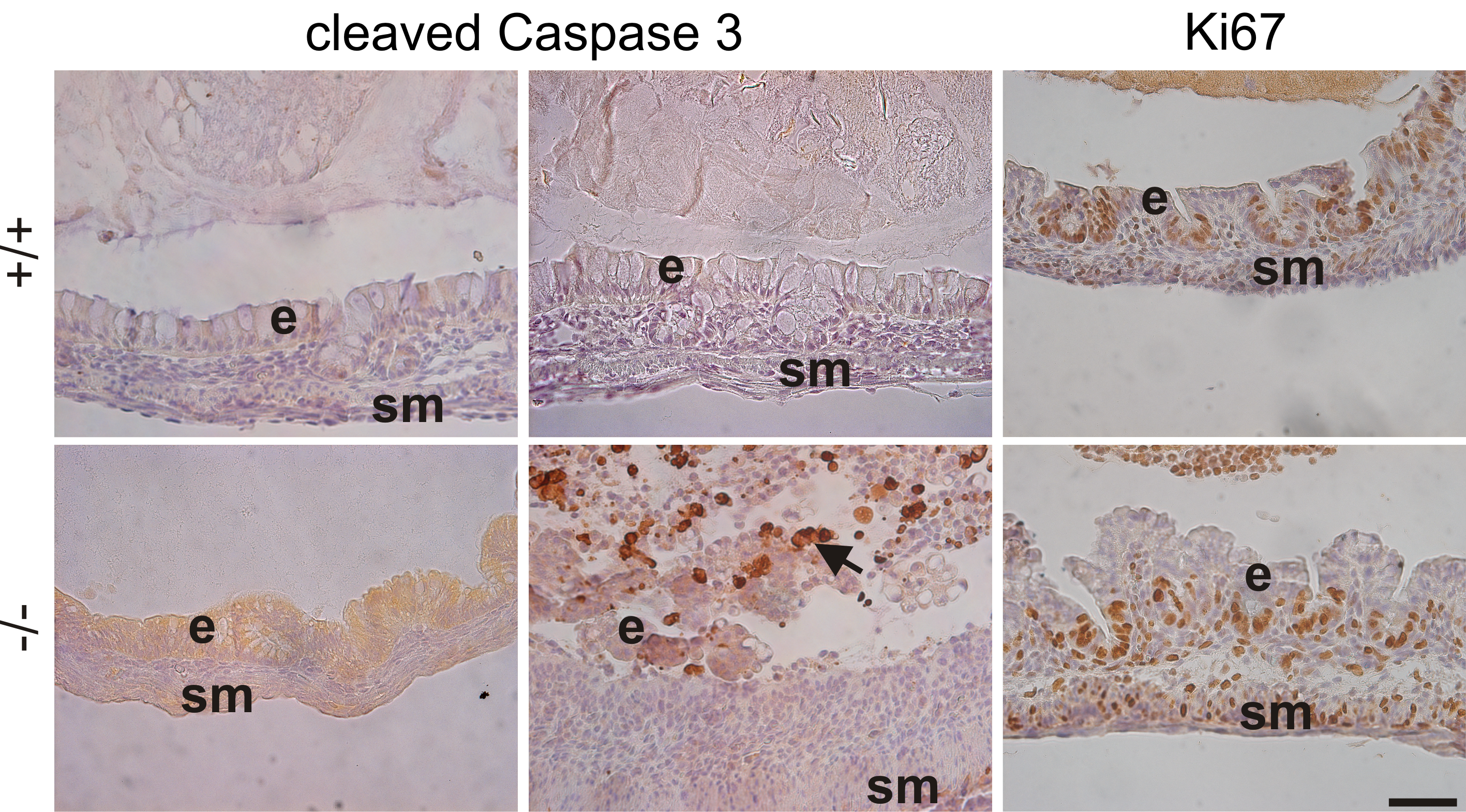

Supplement: Figure S9 — Normal IEC proliferation but detachment induced apoptosis . P1 colons from wild type and Kindlin-1−/− mice were DAB stained for cleaved Caspase-3 to determine apoptosis and Ki67 stained to show proliferating IECs. Apoptosis occurs in detached epithelium of Kindlin-1−/− mice. In areas of still adhering epithelium the number of proliferating IECs is similar between wild type and Kindlin-1−/− mice. Scale bar indicates 50 µm. (8.7 MB TIF) [file pgen.1000289.s009.tif]

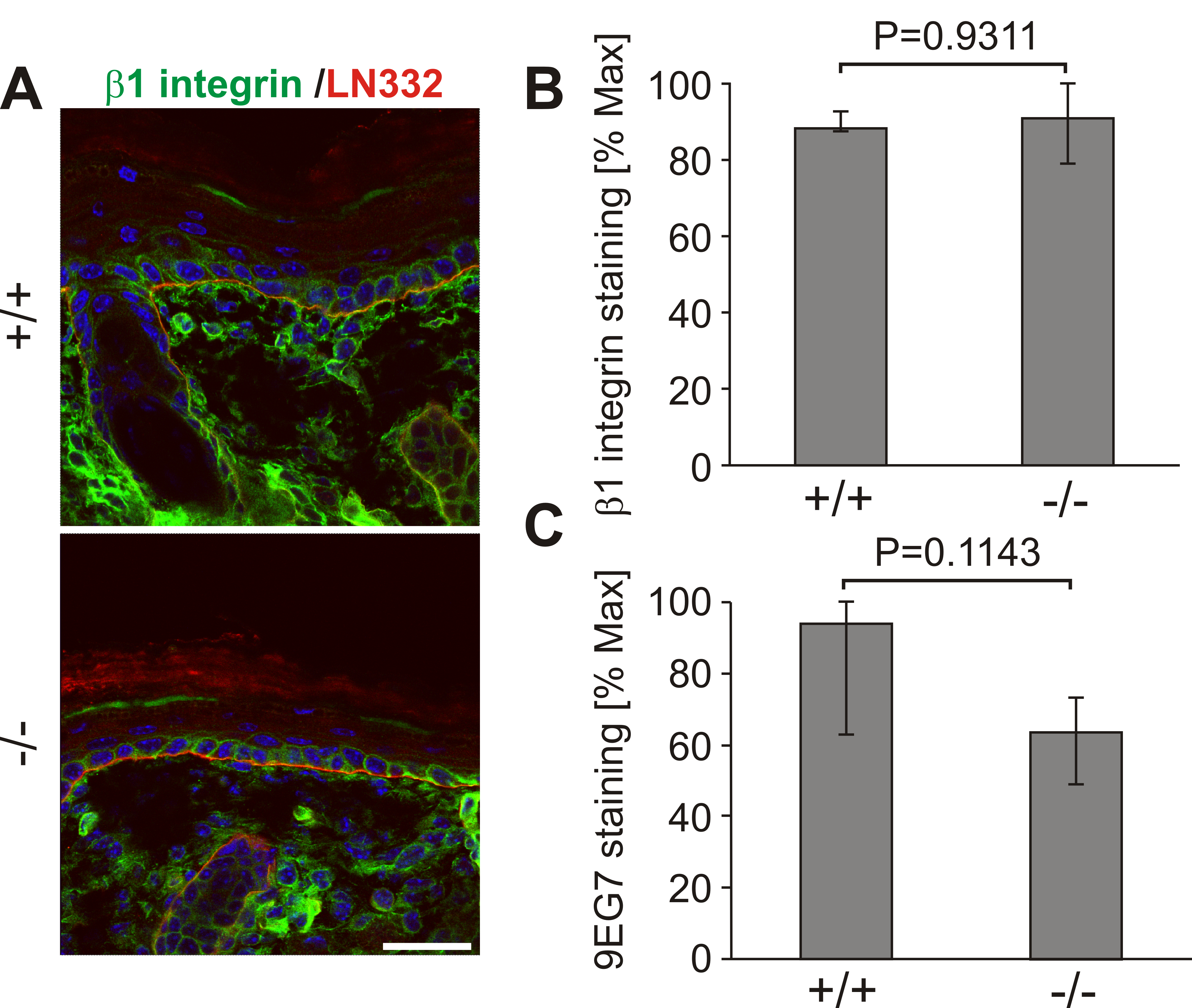

Supplement: Figure S10 — β1 integrin activation in Kindlin-1−/− keratinocytes. (A) Immunofluorescence staining for β1 integrin (green) and Laminin-332 (LN332; red) from P3 backskin shows normal localization of β1 integrin in Kindlin-1−/− backskin. (B) FACS quantification of β1 integrin expression of freshly isolated control and Kindlin-1−/− keratinocytes at P2 shows unaltered β1 integrin expression on basal keratinocytes. Error bars show range (n = 4) (C) 9EG7 FACS quantification of these keratinocytes shows no significant reduction in β1 integrin activation. Error bars show range (n = 4). (8.0 MB TIF) [file pgen.1000289.s010.tif]

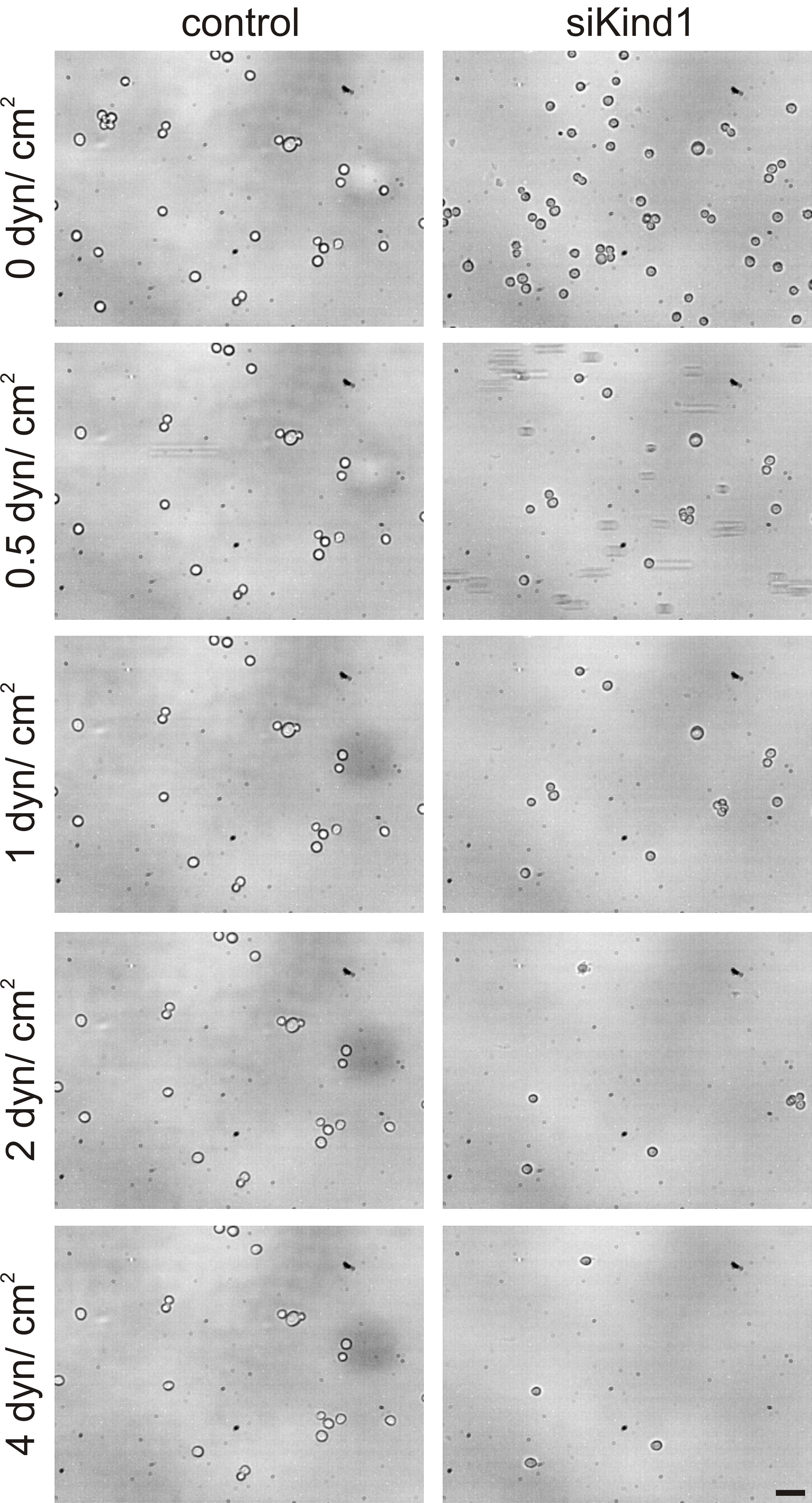

Supplement: Figure S11 — Shear induced detachment of Kindlin-1 depleted HT-29 cells. Control and Kindlin-1 depleted HT-29 (siKind1) cells were plated on Fibronectin-coated flow chamber slides and exposed to increasing shear forces as indicated in the figure. Control cells did not detach from the matrix while Kindlin-1 depleted cells were unable to resist low or high shear forces (compare lane 1 (0dyn/cm2) with lane 2 (0,5dyn/cm2). Scale bar indicates 50 µm. (9.0 MB TIF) [file pgen.1000289.s011.tif]
